# Nanostructure-Engineered Optical and Electrochemical Biosensing Toward Food Safety Assurance

**DOI:** 10.3390/foods14173021

**Published:** 2025-08-28

**Authors:** Xinxin Wu, Zhecong Yuan, Shujie Gao, Xinai Zhang, Hany S. El-Mesery, Wenjie Lu, Xiaoli Dai, Rongjin Xu

**Affiliations:** 1 School of Food and Biological Engineering, Jiangsu University, Zhenjiang 212013, China; 2 School of Energy and Power Engineering, Jiangsu University, Zhenjiang 212013, China

**Keywords:** biosensing, nanostructures, signal amplification, food safety, contaminant detection

## Abstract

Considering the necessity of food safety testing, various biosensors have been developed based on biological elements (e.g., antibodies, aptamers), chemical elements (e.g., molecularly imprinted polymers), physical elements (e.g., nanopores) as recognition substances. According to the sensing patterns of signal transduction, the biosensors could be classified into optical and electrochemical biosensing, including fluorescence sensing, Raman sensing, colorimetric sensing, electrochemical sensing, etc. To enhance the sensing sensitivity, kinds of nanomaterials have been applied for signal amplification. With merits of high selectivity, sensitivity, and accuracy, the sensing strategies have been widely applied for food safety testing. This review highlights their signal output behavior, (e.g., fluorescence intensity shifts, Raman peak alterations, colorimetric changes, electrochemical current/voltage/impedance variations), nanostructure-mediated amplification mechanisms, and the fundamental recognition principles. Future efforts should prioritize multiplexed assay platforms, integration with microfluidics and smart devices, novel biorecognition elements, and sustainable manufacturing. Emerging synergies between biosensors and AI-driven data analytics promise intelligent monitoring systems for predictive food safety management, addressing challenges in food matrix compatibility and real-time hazard identification.

## 1. Introduction

Ensuring food safety—defined as the state and process in which food, throughout its entire lifecycle of production, processing, storage, transportation, and sale, is free from physical, chemical, or biological hazards endangering human health, complies with statutory safety standards, and ensures uncompromised consumer health—remains a global priority for safeguarding public health and maintaining confidence in food supply systems. The persistent threats posed by diverse contaminants [1]—including representative pesticide residues [2] such as organophosphates, veterinary drug [3] residues exemplified by β-lactam antibiotics [4], pathogenic microorganisms [5] like Salmonella spp. and Listeria [6,7], heavy metals [8] including lead(II) and cadmium(II) ions (Pb^2+^ and Cd^2+^) [8], biological toxins such as aflatoxin B1 [9], and illegal additives [10] like Sudan dyes [11]—demand robust analytical frameworks to mitigate risks across farm-to-fork chains [12]. Conventional analytical techniques, such as high-performance liquid chromatography (HPLC) [13], gas chromatography–mass spectrometry (GC–MS) [13], enzyme-linked immunosorbent assays (ELISA) [14], and microbiological culturing [15], have long served as gold standards due to their unparalleled accuracy and sensitivity. Nevertheless, these methods are constrained by time-consuming protocols (e.g., multi-step extraction/purification), specialized infrastructure requirements, and limited adaptability to on-site or high-throughput screening scenarios, which hinder their utility in rapid outbreak containment and real-time monitoring [16]. The inherent limitations of conventional methods drive the urgent development of rapid analytical technologies. This review comprehensively summarizes research advances over the past 15 years.

**Figure 1:**
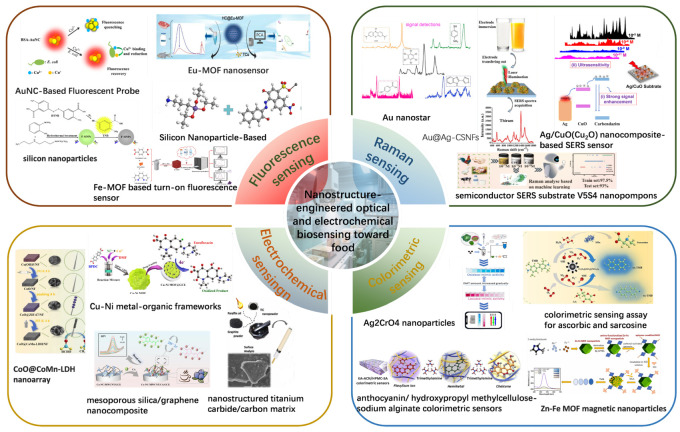
Schematic diagram of nanostructure-engineered biosensing toward food.

The imperative for rapid detection [17,18] technologies has catalyzed innovations in point-of-need analytical platforms. Emerging solutions immunochromatographic lateral flow assays (LFAs) [19], portable biosensors [20], and miniaturized molecular diagnostics [21] such as isothermal amplification and CRISPR-Cas—an adaptive immune system in prokaryotes-like bacteria that recognizes and cleaves foreign genetic material, repurposed as a high-precision genome-editing tool—prioritize user-friendliness, portability, and rapid turnaround times, enabling timely decision-making in decentralized settings. Biosensors [22], in particular, represent a transformative paradigm by integrating biorecognition elements [23] (e.g., antibodies [24], aptamers [25], molecularly imprinted polymers [26]) with transduction mechanisms [27] (e.g., optical, electrochemical) to convert target-analyte interactions into quantifiable electrical or optical signals. Recent advances in nanotechnology have further revolutionized biosensor design: functional nanomaterials [28], including gold nanoparticles (AuNPs) [29], quantum dots (QDs) [30], graphene oxide (GO) [31], and metal–organic frameworks (MOFs) [32], are engineered to amplify signals via mechanisms such as localized surface plasmon resonance (LSPR) [33], Forster resonance energy transfer (FRET) [33], nanozyme catalysis, and enhanced electron transfer efficiency [34] (Figure 1).

Optical [35] and electrochemical [36] biosensing modalities dominate current research trajectories. Optical biosensors leverage fluorescence intensity shifts (e.g., QD-based probes) [37], surface-enhanced Raman scattering (SERS) [38], or colorimetric responses (e.g., AuNP aggregation) [39] for label-free detection. Electrochemical biosensors [40], conversely, exploit current, voltage, or impedance changes induced by redox reactions [41] (e.g., horseradish peroxidase (HRP)-tagged immunoassays) [42]. To address the sensitivity limitations of conventional assays, nanomaterial-mediated signal amplification strategies [43] are systematically tailored to enhance limit of detection (LOD) values into the femtomolar (fM) or even attomolar (aM) range (Table 1).

This review critically examines the operational principles, technological innovations, and practical applications of advanced biosensing platforms in food safety contexts. We elucidate signal transduction mechanisms across optical and electrochemical systems, analyze nanomaterial design rationales for sensitivity enhancement, and dissect biorecognition element–analyte interaction dynamics. Furthermore, we explore emerging trends such as multiplexed detection arrays, AI-integrated smart sensors, and CRISPR-Cas-powered biosensing systems, which collectively herald a new era of predictive and intelligent food safety management.

## 2. Fluorescence Sensing

### 2.1. Signal Output Behavior

Fluorescence sensing encompasses five primary signal output modalities [72,73]—intensity-based [74], wavelength-based [75], time-resolved [76], polarization-based [77], and imaging-based [78,79]—each leveraging distinct signal transduction mechanisms to enhance detection performance. Intensity-based outputs rely on fluorescence enhancement (turn-on) or quenching (turn-off) for rapid response [80,81,82], as seen in Hg^2+^-induced quenching of gold nanoclusters for heavy metal detection with a 2 nM LOD. However, they remain susceptible to environmental interference. Wavelength-based outputs utilize either emission peak shifts [83], such as blue or red shifts caused by changes in the probe’s electronic environment, or employ ratiometric fluorescence [84]—for example, dual-emission ratios in CdSe/ZnS-gold nanoparticle systems that detect bisphenol A at 0.1 μg/L to cancel noise [85]. This approach significantly improves reliability. Time-resolved outputs exploit fluorescence lifetime (τ) changes; long-lifetime probes like Eu^3+^ complexes (τ > 1 ms) [86,87] combined with pulsed excitation eliminate short-lived background fluorescence. A representative application is Tb^3+^-melamine complexes detecting melamine in milk at 0.3 ng/mL via lifetime shifts [88,89]. Polarization-based outputs measure fluorescence polarization alterations, which reflect molecular rotation rates [90], enabling homogeneous rapid screening—exemplified by aflatoxin B1 binding increasing fluorescence polarization by 50%. Imaging-based outputs provide spatial resolution through confocal microscopy or array imaging for target visualization, such as quantum dot-encoded probe arrays generating fluorescence fingerprints to differentiate pathogens [91,92]. To enhance anti-interference capabilities, hybrid strategies are gaining traction; a representative case is ratiometric-time-resolved fusion for multiplex toxin analysis in complex food matrices [93,94,95]. Innovations like smartphone-assisted readouts, deep learning-enhanced imaging, and wearable fluorescent patches are driving the field toward on-site rapid detection, intelligent analysis, and portability, collectively advancing food safety monitoring precision and practicality [96,97,98].

### 2.2. Fluorescence Sensing Leverages Diverse Nanomaterials

**Figure 2:**
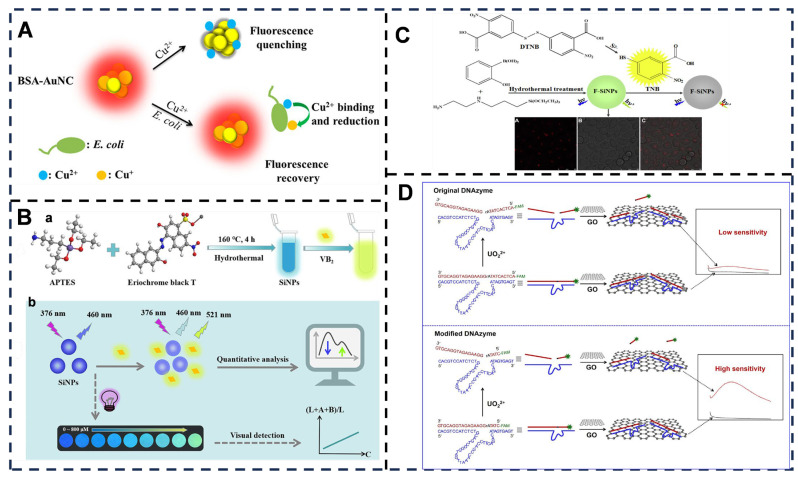
(**A**) Schematic illustration of the working principle of the on-off-on AuNC-based fluorescent probe for rapid *E. coli* differentiation, detection, and bactericide screening. Adapted with permission from Ref. [44]. Copyright 2018 American Chemical Society. (**B**) Synthesis of the SiNPs (**a**) and dual-platform detection of vitamin B_2_ by fluorescence and visualization methods (**b**). Adapted with permission from Ref. [45]. Copyright 2023 American Chemical Society. (**C**) Schematic illustration of preparation of F-SiNPs and their applications for sensing S^2−^ and cell imaging. Adapted with permission from Ref. [46]. Copyright 2022 Elsevier. (**D**) Schematic representation of the DNAzyme/GO nanosystem for turn-on sensing of UO_2_^2+^. Adapted with permission from Ref.[47]. Copyright 2022 Elsevier.

Fluorescence sensing leverages diverse nanomaterials: Noble metal nanomaterials include bovine serum albumin (BSA)-stabilized gold nanoclusters (Au NCs)-based “on-off-on” probes for *E. coli* detection [99,100], where Cu^2+^ initially quenches fluorescence via coordination with BSA amino acids, but bacterial presence reduces and removes Cu^2+^, restoring fluorescence. The innovation of this method lies in leveraging *E. coli*’s unique copper metabolism pathway to achieve specific detection (LOD = 89 CFU/mL). It avoids complex labeling procedures and enables detection within 0.5 h along with antimicrobial susceptibility testing within just 2 h—significantly faster than traditional culture or PCR-based methods which typically require 24 to 48 h. However, its effectiveness is highly specific to *E. coli* and cannot differentiate between bacterial subtypes such as drug-resistant and non-resistant strains [44,101] (Figure 2A). Semiconductor quantum dots such as fluorescent silicon nanoparticles (SiNPs) [102], synthesized hydrothermally from 3-aminopropyltriethoxysilane and Eriochrome Black T, offer water solubility, thermal stability [103], pH tolerance, and photobleaching resistance for ratiometric riboflavin (VB_2_) detection [104]—VB_2_ quenches the 460 nm emission while generating a new peak at 521 nm. This SiNPs-based fluorescence method achieves a linear detection range of 0.5–60 μM for VB_2_ with a 135 nM limit of detection. Compared to conventional AgNPs colorimetry—which exhibits similar sensitivity (LOD = 167 nM) but a substantially narrower dynamic range of 0.17–4.67 μM—and carbon dot fluorescence demonstrating higher sensitivity (LOD = 37.2 nM) albeit with a narrower linear range of 0.35–35.9 μM, the SiNPs approach maintains high sensitivity across an extended concentration span. This broad linear response makes it particularly suitable for quantifying variable VB_2_ concentrations in complex real-world samples. Furthermore, SiNPs demonstrate superior environmental stability over organic fluorophores like photodegradation-prone carbon dots, exhibiting tolerance to temperature variations (5–85 °C), wide pH conditions (4–10), high ionic strength environments (≤100 mM NaCl), and prolonged light exposure [45] (Figure 2B). Yellow-green emitting fluorescent silicon nanoparticles (F-SiNPs) synthesized via a one-step hydrothermal method function as highly selective and sensitive fluorescence sensors for sulfide ions (S^2−^), enabling detection in tap water and lake water samples. This approach addresses key limitations of conventional fluorescent nanomaterials—such as high toxicity and elevated costs associated with heavy-metal-containing quantum dots and noble metal nanoclusters—while maintaining robust sensing performance [46,105] (Figure 2C). Upconversion nanoparticles (UCNPs) [106] show great promise for diverse applications due to their superior properties, including a large anti-Stokes shift and excellent photostability and chemical stability. Leveraging UCNPs and UO_2_^2+^-specific DNAzymes, two label-free upconversion fluorescence sensing platforms were developed for the sensitive quantitative detection of UO_2_^2+^. An intelligent nanosystem integrating DNAzymes and graphene oxide was constructed to overcome the limitations of conventional uranyl ion detection methods (such as mass spectrometry and atomic absorption spectroscopy), which often involve bulky instrumentation and complex procedures. This system achieves a low LOD of 25 pM and exhibits a wide dynamic range of 0.1–40 nM. When tested against 24 metal ions—including common interferents such as Cu^2+^, Pb^2+^, and Hg^2+^—only UO_2_^2+^ triggered a significant signal response, demonstrating high selectivity. Future research could integrate smartphone-based fluorescence readout devices to enable rapid on-site detection [47] (Figure 2D).

**Figure 3:**
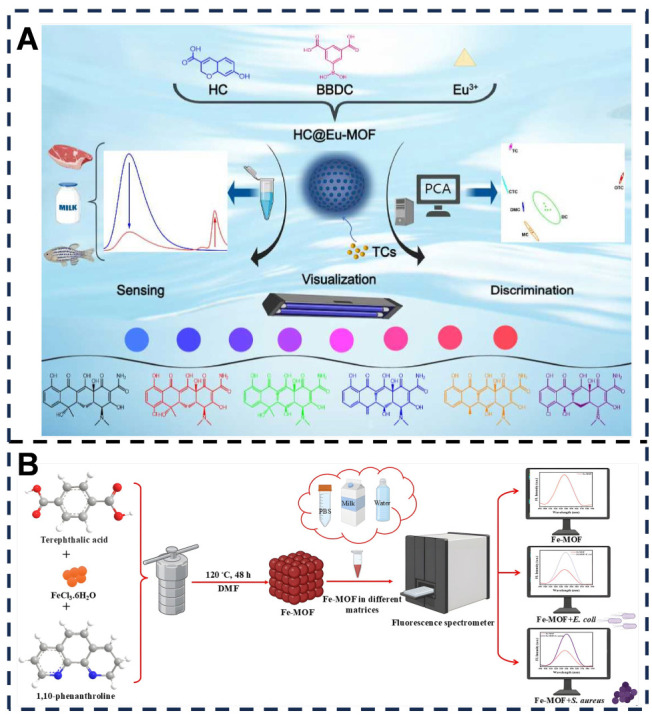
(**A**) The synthesis and application of HC@Eu-MOF nanosensor for sensing and discrimination of TCs. Adapted with permission from Ref. [48]. Copyright 2025 Elsevier. (**B**) Fe-MOF based turn-on fluorescence sensor for the rapid detection of foodborne pathogens in multiple matrices. Adapted with permission from Ref. [49]. Copyright 2022 Elsevier.

Metal–organic frameworks (MOFs) demonstrate versatile sensing capabilities: A europium-based MOF embedded with 7-hydroxycoumarin-3-carboxylic acid (HC@Eu-MOF) efficiently detects tetracycline analogues (TCs) [107]. TCs trigger opposite-direction fluorescence changes at dual wavelengths via synergistic inner filter effect [108] and antenna effect (AE) [109], yielding stable ratiometric signals within 90 s. Principal component analysis [110] enables 100% accurate discrimination of six TCs—chlortetracycline (CTC), oxytetracycline (OTC), tetracycline (TC), metacycline (MC), doxycycline (DC), and demeclocycline—in complex mixtures. This method offers the advantages of rapid response and high sensitivity, enabling detection within 90 s. The LOD for OTC was as low as 4.8 nM, while the LODs for other TCs (TC, CTC, etc.) ranged from 16.5 to 56.4 nM. However, sample pretreatment (e.g., centrifugation/filtration), similar to that required for HPLC, remains necessary for real samples. Overall, this study addresses a gap in traditional methods by providing rapid on-site analysis with multi-component discrimination capabilities. It offers an innovative solution combining laboratory-grade accuracy with on-site convenience for food safety and environmental monitoring applications [48] (Figure 3A). Complementarily, a solvothermally synthesized iron MOF (Fe-MOF), characterized by fourier-transform infrared spectroscopy (FTIR), X-ray diffraction (XRD), scanning electron microscopy with energy-dispersive X-ray spectroscopy (SEM-EDX), and thermogravimetric analysis (TGA), serves as a “fluorescence turn-on” biosensor for rapid detection of foodborne pathogens (*Staphylococcus aureus*, *Escherichia coli*). Bacterial surface components modulate electron transfer, enhancing fluorescence. Evaluated in Phosphate-buffered saline (PBS), drinking water, and milk, the sensor achieves LOD of 0.464 log CFU/mL for *S. aureus* and 0.584 log CFU/mL for *E. coli* within 1 h, with 95–104% spiked recoveries confirming practical utility. This method overcomes the limitation of conventional sensors restricted to buffer testing, enabling reliable detection in milk. It leverages the inherent fluorescence of Fe-MOF, eliminating the need for complex functionalization with antibodies or nucleic acid probes. Future improvements could enhance its capability to detect a broader spectrum of pathogens, including Salmonella, and facilitate the development of integrated devices, such as microfluidic chips coupled with smartphone-based fluorescence detection [49] (Figure 3B).

### 2.3. Fundamental Recognition Principles

Fluorescence sensing relies on fundamental recognition principles that trigger signal changes through specific molecular interactions [111,112,113]. Key mechanisms include bioaffinity recognition, where antibody–antigen binding enables target locking—exemplified by quantum dot-labeled antibodies achieving 0.01 ng/mL aflatoxin detection via Förster resonance energy transfer (FRET) quenching [114]—or aptamer conformational switching such as thrombin-induced unfolding separating fluorophores from quenchers [115]. Molecularly imprinted polymers (MIPs) leverage template cavities for emission red-shifts upon tetracycline entry [116] or competitive displacement, achieving 1.2 ng/mL tetracycline detection in milk [117]. Host–guest chemistry exploits cyclodextrin hydrophobic encapsulation for cholesterol sensing through pyrene excimer formation [118], utilizes sulfonated calix [4] arene ion selectivity to achieve 0.05 nM UO_2_^2+^ detection via dequenching [119], and employs cucurbituril (CB) confinement enhancing amantadine fluorescence quantum yield to a 0.8 ng/g detection limit [120]. Chemically driven interactions encompass Zn^2+^-Zinpyr-1 chelation amplifying fluorescence 50-fold [121], maleimide-thiol covalent adducts activating toxin detection [122], and π-π stacking-mediated pyrene-benzo[a]pyrene ratiometric signaling for 0.02 μg/kg polycyclic aromatic hydrocarbons (PAHs) detection [123]. Stimuli-responsive recognition employs pH-sensitive FITC protonation quenching for amine monitoring [124,125], enzymatic β-galactosidase substrate cleavage releasing fluorescein with 100× amplification for *E. coli* [126], and photochromic azobenzene isomerization modulating FRET efficiency. These mechanisms form synergistic recognition-to-signal-transduction cascades: bioaffinity interactions with dissociation constants below 10^−9^ M excel for macromolecules [127]; molecularly imprinted polymers and host–guest systems resist matrix interference; coordination chemistry enables second-scale ion responses, such as Zn^2+^ detection at 5 nM LOD [128,129]. Emerging paradigms like CRISPR-Cas dynamic recognition through reporter cleavage for viral targets [130] and machine learning-enhanced olfactory arrays using porphyrin probes for volatile organic compounds (VOCs) discrimination [131,132], are driving evolution toward multi-analyte screening networks for comprehensive food safety assessment.

## 3. Raman Sensing

### 3.1. Signal Output Behavior

Raman sensing deciphers molecular vibrational fingerprints for target detection through five core signal output modalities. Peak shift manifests as spectral displacement, exemplified by aflatoxin B1 inducing a 30 cm^−1^ red-shift in DNA backbone vibration from 1090 to 1120 cm^−1^ [133,134]. Intensity variation includes direct signal quantification where melamine concentration correlates linearly with 702 cm^−1^ peak intensity at 0.1 ppm detection limit [135,136], alongside surface-enhanced Raman scattering employing noble metal nano-hotspots to amplify signals by 10^6^–10^14^ fold—gold nanostar dimers exemplify this by detecting chlorpyrifos down to 0.01 ppb [137,138]. Linewidth broadening reflects molecular disorder changes, such as lipid oxidation expanding the C=C stretching peak’s full-width half-maximum from 15 to 28 cm^−1^ [139]. Imaging analysis encompasses confocal Raman chemical mapping for spatial pesticide distribution profiling on agricultural produce and stimulated Raman scattering-based 3D reconstruction for antibiotic penetration depth quantification [133,140,141]. Multimodal fusion integrates complementary techniques like SERS-colorimetric dual-output for visual pre-screening with Raman validation, and electrochemistry-SERS coupling achieving dopamine detection at 0.1 nM sensitivity [142,143,144]. Contemporary advancements harness deep learning for sub-0.5 cm^−1^ peak shift resolution via convolutional neural network (CNN) algorithms [145], smartphone-integrated portable SERS platforms enabling on-site aflatoxin detection at 0.05 ppb, and millisecond-fast Raman dynamic tracking. These innovations overcome matrix interference and portability constraints in food safety, driving applications including rapid pesticide screening at 0.1 ppb organophosphorus detection limits and pathogen classification exceeding 98% accuracy through imaging-machine learning integration—collectively advancing intelligent field-deployable diagnostics [145,146,147].

### 3.2. Raman Sensing Leverages Diverse Nanomaterials

**Figure 4:**
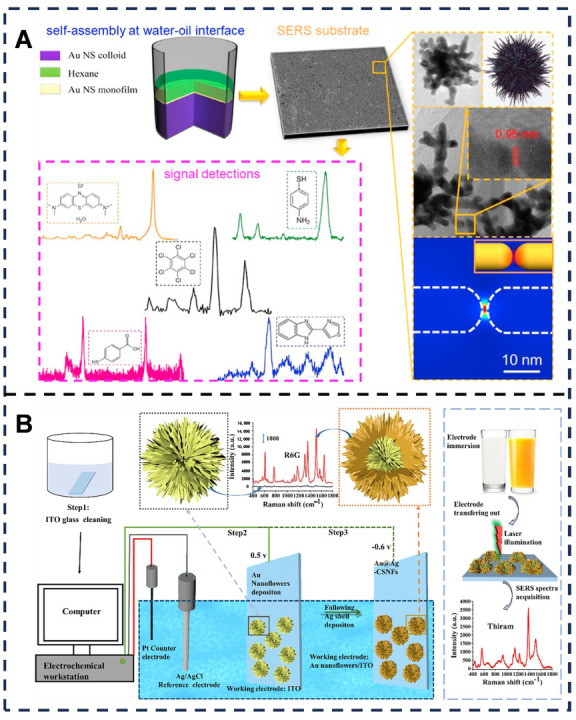
(**A**) Tip-to-tip assembly of urchin-like Au nanostar at water–oil interface for surface-enhanced Raman spectroscopy detection. Adapted with permission from Ref. [50]. Copyright 2021 Elsevier. (**B**) The illustration outlines the synthesis of Au@Ag-CSNFs, and its use as a SERS substrate for thiram detection in foodstuff. Adapted with permission from Ref. [51]. Copyright 2023 Elsevier.

In the field of Raman sensing concerning noble metal nanomaterials, Cong et al. [50] (Figure 4A) fabricated a large-area gold nanostar (NS) monolayer film using an oil–water interface self-assembly method. This Au NS monolayer contains numerous “hot spots” [148] both at the tips of the Au NSs and between adjacent tips, which enhance the local electromagnetic field and generate strong SERS signals, enabling the detection of molecules such as methylene blue and thiabendazole. This method, utilizing Au NS as the substrate, achieves ultra-high sensitivity, with an LOD for Rhodamine 6G reaching 4.2 × 10^−12^ M. This significantly outperforms traditional methods based on lithographic nanoarrays. Furthermore, the method exhibits superior versatility compared to conventional approaches. It eliminates the need for substrate modification specific to different target molecules and successfully detects diverse analytes, including dyes, pesticides, environmental toxins, and thiol compounds, demonstrating its multi-analyte detection capability. Wang et al. [51] (Figure 4B) synthesized an innovative bimetallic gold–silver core–shell nanoflower (Au@Ag-CSNFs) SERS substrate on indium tin oxide (ITO) glass via electrochemical deposition. The Au@Ag-CSNFs possess a unique structure with uniformly distributed gaps and cavities between each branch, serving as potential target capture sites and “hot spots.” This substrate exhibits strong stability and reproducibility, allowing for the sensitive detection of thiram in complex food matrices like milk and juice [149]. This method exhibits high specificity for thiram detection, selectively adsorbing the target via Ag-S bonds to avoid interference from other species. It also demonstrates a wide linear range from 1.0 × 10^−7^ to 1.0 × 10^−3^ mol/L and an ultra-low LOD of 7.09 × 10^−8^ mol/L. Substrate fabrication is completed within just 3 min, requiring no templates or complex equipment. However, limitations include challenges in substrate reusability, the relatively high cost associated with the use of precious metals such as gold and silver, unsuitability for large-scale screening, and the need for re-optimization of the adsorption interface to detect other contaminants beyond thiram.

**Figure 5:**
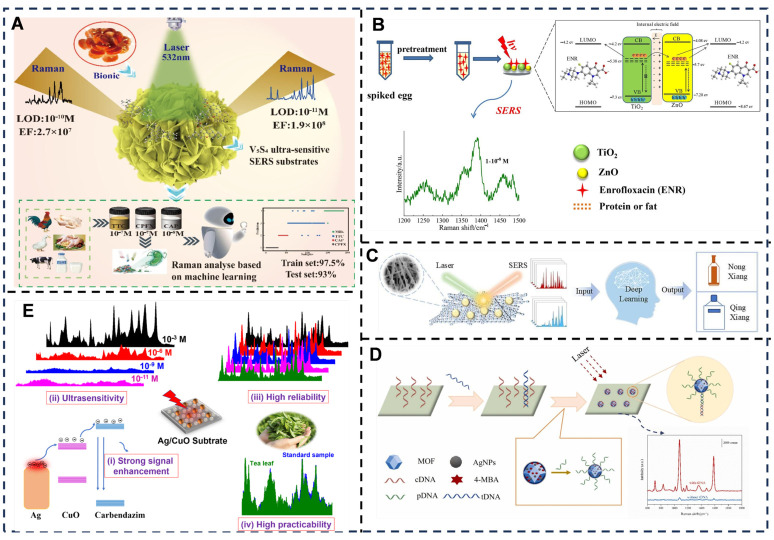
(**A**) Schematic illustration of V5S4 NPPs SERS detection and identification of different antibiotics using machine learning. Adapted with permission from Ref. [52]. Copyright 2024 Elsevier. (**B**) A new semiconductor heterojunction SERS substrate for ultra-sensitive detection of antibiotic residues in egg. Adapted with permission from Ref. [53]. Copyright 2024 Elsevier. (**C**) Optoplasmonic MOF film for SERS-AI detection of flavor from Baijiu. Adapted with permission from Ref. [54]. Copyright 2025 Elsevier. (**D**) A novel AgNPs/MOF substrate-based SERS sensor for high-sensitive on-site detection of wheat gluten. Adapted with permission from Ref. [55]. Copyright 2025 Royal Society of Chemistry. (**E**) Ultrasensitive detection of carbendazim pesticide in tea leaves using a green Ag/CuO(Cu_2_O) nanocomposite-based SERS sensor: role of metal/semiconductor transition in sensing performance. Adapted with permission from Ref. [56]. Copyright 2023 Elsevier.

Regarding semiconductor materials, vanadium sulfide nanopompon (V_5_S_4_ NPPs) as a substrate, combined with a newly developed convolutional residual neural network AI processing method, achieved highly sensitive and specific detection of three common antibiotics—ciprofloxacin, tetracycline, and chloromycin (with LOD of 10^−7^ M, 10^−8^ M, and 10^−7^ M, respectively)—and precise identification with 97.5% accuracy. The SERS enhancement mechanism of this method arises from a dual-resonance synergistic effect comprising photoinduced charge transfer resonance and molecular resonance, coupled with weak electromagnetic enhancement, while chemical enhancement serves as the dominant mechanism. This approach delivers exceptional sensitivity and specificity, whereby the chemical enhancement mechanism selectively amplifies antibiotic signals while suppressing interference from proteins and fats in milk. Furthermore, the substrate maintains structural stability under harsh conditions including strong alkali, physiological environments, and low temperatures, surpassing the performance of precious metal substrates in these aspects. However, structural stability diminishes in acidic environments, rendering the nanosheets susceptible to corrosion. Compared to traditional precious metal substrates, this method additionally offers advantages of lower cost and scalability [52] (Figure 5A). This provides an efficient method for monitoring antibiotic residues in milk, significantly enhancing food safety. A titanium dioxide/zinc oxide (TiO_2_/ZnO) heterostructure substrate achieved multi-dimensional regulation of charge carrier behavior within the substrate through strong interfacial coupling effects, significantly improving charge transfer efficiency between the substrate and molecules. In practical antibiotic residue detection in eggs, this substrate demonstrated ultra-high sensitivity (with LOD of 3.94 × 10^−8^ M) and good reproducibility and repeatability. This method enables simultaneous multi-component detection by leveraging the fingerprint characteristics of SERS spectra to differentiate residues from either the same or different classes of antibiotics. In contrast, conventional techniques such as chromatography and mass spectrometry typically require complex separation procedures and face challenges in achieving rapid synchronous multi-analyte analysis. Furthermore, the TiO_2_/ZnO semiconductor substrate offers significant cost advantages due to its low-cost materials while demonstrating robust stability, evidenced by signal attenuation of less than 5% after two months of ambient storage and strong resistance to acid corrosion [53] (Figure 5B). MOF materials, composite films like MOF-semi-encapsulated silver nanowires (AgNWs@MOF) combined with silica microspheres, achieved dual functions of efficient gas adsorption and SERS enhancement [150]. An artificial neural network (ANN) algorithm was constructed for liquor flavor and brand recognition with nearly 90% accuracy. This method leverages the inherent porosity of MOFs to efficiently adsorb gaseous flavor molecules, overcoming challenges posed by low gas density and rapid diffusion. For liquid-phase detection, it achieves a remarkably low LOD of 10^−13^ mol/L for the model molecule PATP, representing a 2–3 order of magnitude improvement over conventional SERS techniques. In gas-phase detection, the LOD for PATP vapor reaches 10^−7^ mol/L. However, limitations include susceptibility of AgNWs to oxidation potentially causing signal degradation during prolonged use, signal instability arising from fluctuating adsorption kinetics of gas molecules, and higher fabrication costs of AgNW-MOF substrates compared to conventional platforms. Future development should focus on implementing oxidation-resistant coatings such as graphene encapsulation, optimizing MOF pore-size distribution to enhance adsorption equilibrium, and exploring cost-effective alternative materials like CuNWs [54] (Figure 5C). A surface-enhanced Raman scattering (SERS) sensor based on a silver nanoparticle/MOF (AgNPs/MOF) substrate was used for highly sensitive on-site detection of wheat gluten. The detection process occurred on a novel in situ synthesized AgNPs/MOF-modified SERS substrate with an enhancement factor of 1.89 × 10^5^. This sensor demonstrated exceptional sensitivity with a broad detection range spanning from 1 × 10^−15^ to 2 × 10^−6^ mol/L and achieved an ultra-low LOD of 1.16 × 10^−16^ mol/L. This performance enables matrix-interference-free monitoring of trace wheat gluten in complex food systems [55]. This method exhibits exceptional anti-interference capability, where complex matrices in deeply processed foods such as soy sauce and vinegar—including sugars and fats—do not compromise detection accuracy, overcoming the matrix susceptibility common in conventional approaches. It employs a dual-layer specificity mechanism: (1) DNA recognition via wheat-gluten-specific tDNA fragments, and (2) a sandwich-type assembly that reduces non-specific binding through dual hybridization. However, limitations include prolonged detection time requiring 150 min for hybridization steps (30 min for tDNA binding plus 120 min for SERS probe assembly), dependence on DNA extraction that may cause missed detection in DNA-degraded samples, and elevated costs from MOF synthesis and DNA probe modification. Future improvements should focus on accelerating hybridization kinetics, developing direct gluten protein detection strategies, and implementing batch substrate production to reduce costs (Figure 5D). Concerning composite nanomaterials, a silver/copper oxide (Ag/CuO) nanocomposite substrate demonstrated ultra-sensitivity for detecting the widely used harmful pesticide carbendazim (CBZ) in tea, achieving a detection limit as low as 8.85 × 10^−11^ M, significantly surpassing that of bare silver substrates (10^−6^ M). Its high reliability was confirmed by excellent reproducibility (relative standard deviation < 10%). Practical spiked recovery rates of CBZ in fresh tea leaf samples reached 85–106%, fully validating its utility. The enhancement mechanism primarily relies on the synergistic metal/semiconductor effect, wherein silver nanoparticles provide electromagnetic enhancement while copper oxide facilitates chemical enhancement. Crucially, strategic energy-level alignment enables efficient charge transfer: the conduction band minimum of CuO (−3.86 eV) resides between the Fermi level of Ag (−4.26 eV) and the LUMO of carbendazim (−3.56 eV), establishing an “energy bridge” that significantly reduces charge-transfer barriers. This contrasts with the less favorable energy position of Cu_2_O (−4.19 eV). The approach benefits from green-synthesized Ag/CuO nanocomposites that integrate high sensitivity, stability, and cost-effectiveness. This provides a streamlined solution for pesticide residue detection requiring no complex sample pretreatment while enabling field-deployable analysis, thereby advancing SERS technology from laboratory research toward practical agricultural monitoring [56,151,152] (Figure 5E).

### 3.3. Fundamental Recognition Principles

The fundamental recognition principles in Raman sensing rely on the synergistic integration of molecular vibrational fingerprint uniqueness, nanomaterial enhancement mechanisms [153], and specificity strategies, where unique Raman shifts at characteristic frequencies—such as the 702 cm^−1^ ring-breathing vibration for melamin [154]—serve as irreplicable spectroscopic identifiers. Sensitivity is achieved through dual enhancement: electromagnetic amplification via localized surface plasmon resonance hotspots in sub-5-nm Au nanostar dimer gaps boosting chlorpyrifos signals by 10^9^-fold [155], and chemical charge transfer through band-structure engineering in V_5_S_4_ nanopompons enabling 10^−8^ M tetracycline detection [156,157]. Specificity derives from physical adsorption in MOF porous frameworks concentrating baijiu flavor molecules, chemical targeting with thiol-functionalized substrates capturing wheat gluten down to 10^−16^ M, and biological locking via aptamer-specific aflatoxin binding. Signal interpretation encompasses peak displacement like Hg^2+^-induced 5.8 cm^−1^ G-band redshifts in graphene [158], intensity-based quantification through linear concentration-peak intensity correlations, and AI-driven resolution of overlapping peaks at 97.5% accuracy using CNN algorithms. Future advancements include CRISPR-SERS for single-virus detection [159], NIR-penetrable probes exceeding 5 mm tissue depth, and antifouling coatings, collectively driving recognition precision toward single-molecule resolution to establish ultrasensitive analysis foundations for complex matrices.

## 4. Colorimetric Sensing

### 4.1. Signal Output Behavior

Colorimetric sensing employs five core signal output mechanisms [160], with bulk solution color change enabling absorbance quantification—as exemplified by organophosphorus pesticides inhibiting acetylcholinesterase to induce fading of the yellow color from 5,5′-dithiobis(2-nitrobenzoic acid) (DTNB) reaction products, detectable at 412 nm [161]—alongside visual comparison methods demonstrated in pH test strips [162]; nanomaterial aggregation-induced spectral shifts leverage analyte-triggered LSPR peak displacement, exemplified by gold nanoparticles transitioning from red to blue for 0.1 ng/mL ochratoxin A detection, or silver nanocubes shifting from yellow to brown-red for 5 nM Hg^2+^ sensing [163,164]; microfluidic and lateral flow arrays achieve high-throughput spatial encoding, demonstrated through T/C line intensity ratios quantifying aflatoxin B1 down to 0.05 ppb [165]; ratiometric colorimetry uses dual-signal ratios, such as the absorbance ratio of AuNPs at 520 nm vs. CdTe QDs at 625 nm for 0.1 μM bisphenol A detection or the TMB (3,3′,5,5′-tetramethylbenzidine)/OPD (o-phenylenediamine) dual-enzyme chromogenic ratios for glucose monitoring, to resist interference [166]; smart surface responses utilize stimuli-responsive materials such as PNIPAM-encapsulated gold nanorods exhibiting temperature-driven red-to-blue spectral shifts and porphyrin films transitioning from green to red upon amine exposure for real-time meat spoilage indication [167]. Cutting-edge advancements focus on digital interpretation including smartphone RGB analysis extracting chromatic parameters with under 5% error in pesticide detection, CNN algorithms decoding microfluidic arrays for multiplex toxin identification exceeding 95% accuracy, and wearable colorimetric patches dynamically tracking physiological indicators. These innovations collectively push detection limits to picomolar levels while evolving toward CRISPR-mediated colorimetric outputs—specifically Cas12a cleavage-triggered coloration systems—and IoT-enabled real-time monitoring platforms [168,169].

### 4.2. Colorimetric Sensing Leverages Diverse Nanomaterials

**Figure 6:**
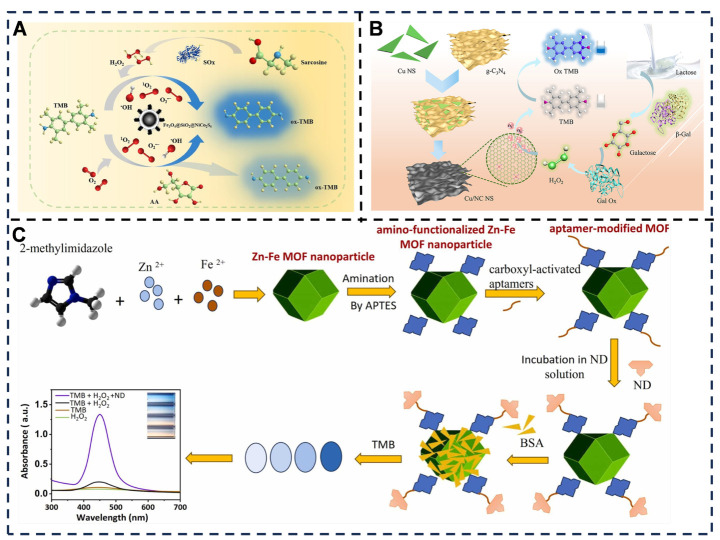
(**A**) Development of a colorimetric sensing assay for ascorbic and sarcosine utilizing the dual-class enzyme activity of Fe_3_O_4_@SiO_2_@NiCo_2_S_4_. Adapted with permission from Ref. [57]. Copyright 2023 Elsevier. (**B**) Cascade reaction biosensor based on Cu/N co-doped two-dimensional carbon-based nanozyme for the detection of lactose and β-galactosidase. Adapted with permission from Ref. [58]. Copyright 2025 Elsevier. (**C**) Schematic illustration of preparation process of Zn-Fe MOF magnetic nanoparticles and the assemble process for the MOF-based aptasensor. Adapted with permission from Ref. [60]. Copyright 2025 Elsevier.

In the field of colorimetric sensing, nanozyme materials have demonstrated significant advancements: Wang et al. [57] synthesized Fe_3_O_4_@SiO_2_@NiCo_2_S_4_ nanocomposites via eutectic solvent-assisted hydrothermal method. Leveraging oxidase-like and peroxidase-like activities, they established colorimetric detection methods for ascorbic acid (AA) (linear range: 1–200 μM, LOD: 0.36 μM) and sarcosine (linear range: 1.25–350 μM, LOD: 0.42 μM). The detection principle for AA relies primarily on the oxidase-like activity of the material, which catalyzes the oxidation of TMB to generate a blue-colored product. Subsequent introduction of AA reduces the oxidized TMB, resulting in color fading. This method enables quantitative analysis of AA in food matrices such as juices and beverages, as well as in serum samples. Compared to traditional approaches characterized by prolonged detection times and laboratory confinement, this method achieves significantly shorter analysis times—requiring only 1 h for AA quantification—while supporting field-deployable detection. It further demonstrates robust stability with high tolerance to ionic strength and broad pH adaptability [170] (Figure 6A). Notably, the nanocomposites achieved high-precision sarcosine quantification in human urine samples. Concurrently, Cu/N-doped carbon-based nanozymes (Cu/NC NS) were synthesized by anchoring Cu atoms on 2D carbon nanosheets, exhibiting exceptional peroxidase-like activity for lactose detection in milk and β-galactose monitoring in human serum [58]. This method operates through a catalytic mechanism where Cu-N_x_ active sites activate H_2_O_2_ to generate superoxide radicals (·O_2_^−^), which subsequently oxidize chromogenic substrates such as TMB. For lactose detection, it exhibits a linear range of 0.1–1.4 mM with a LOD of 0.03 mM. For β-galactosidase detection, the linear range spans 0.025–0.2 U/mL with an LOD of 0.01 U/mL. Key advantages include dual-function integrated detection—enabling simultaneous quantification of both lactose and β-Gal on a single platform through cascade reaction design that prevents cross-interference—along with low-cost operation requiring no expensive instrumentation, and good stability evidenced by nanozyme reusability for at least four cycles. However, limitations comprise stringent reaction conditions demanding precise control of pH and temperature, high nanozyme synthesis costs due to energy-intensive pyrolysis at 800 °C and acid-waste generation during processing, and a relatively narrow dynamic range that fails to cover high-concentration samples such as whey powder (Figure 6B). Regarding metal–organic frameworks, the Ta-MOF substrate mimicked peroxidase activity to catalyze H_2_O_2_-mediated TMB oxidation into blue radicals [171,172], with antioxidants causing solution decolorization—exhibiting apparent Michaelis–Menten constants (K_m_) of 3.180 mM (TMB) and 0.0109 μM (H_2_O_2_), and LODs of 0.19 μM (ferulic acid), 0.06 μM (tannic acid), and 0.11 μM (chlorogenic acid) [59]. A colorimetric aptasensor based on bimetallic Zn-Fe MOF nanostructures was developed and applied for the detection of nandrolone (ND), an anabolic steroid agent used in doping in sports. The sensor was synthesized by functionalizing Zn-Fe MOF nanoparticles with amino groups and conjugating them with carboxyl-activated aptamers specific to ND. Validated by TMB-H_2_O_2_ colorimetric assay, the sensor exhibited excellent selectivity and sensitivity for ND within a concentration range of 0.005–1000 μM, with a LOD of 0.85 nM [60]. Compared to traditional HPLC methods, this approach offers significant advantages including a substantially reduced detection time of approximately 45 min, lower instrumentation requirements, simplified operation, decreased reagent costs due to scalable MOF synthesis, potential development into portable test strips, and an extended linear detection range. However, it is limited by its single-analyte detection capability (Figure 6C).

**Figure 7:**
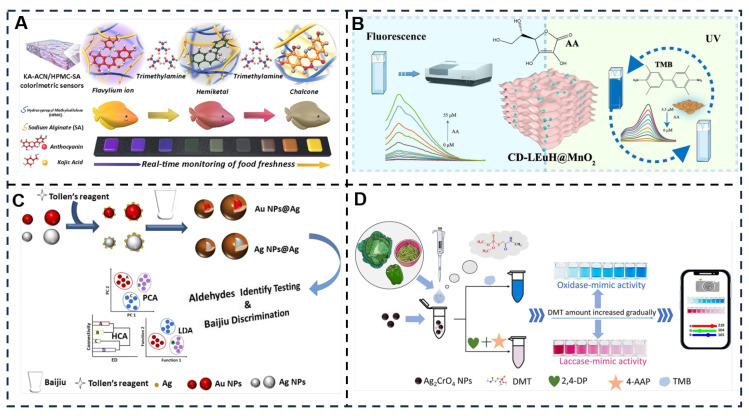
(**A**) Kojic acid-modulated anthocyanin/hydroxypropyl methylcellulose-sodium alginate colorimetric sensors for real-time freshness monitoring of salmon. Adapted with permission from Ref. [61]. Copyright 2025 Elsevier. (**B**) CD-LRH@MnO_2_ fluorescent/colorimetric dual-mode sensor for the detection of ascorbic acid. Adapted with permission from Ref. [62]. Copyright 2025 Elsevier. (**C**) A colorimetric sensor array based on Ag deposition on the surface of Au/Ag nanoparticles for accurately discrimination of Baijiu. Adapted with permission from Ref. [63]. Copyright 2025 Elsevier. (**D**) Bimetallic Ag_2_CrO_4_ nanoparticles with dual-enzyme-mimic activities in colorimetric sensor for sensitive and highly selective detection of dimethoate in vegetables. Adapted with permission from Ref. [64]. Copyright 2025 Elsevier.

Natural pigment-based sensors addressed food freshness monitoring: a kojic acid (KA)-modulated anthocyanin (ACN)/hydroxypropyl methylcellulose (HPMC)-sodium alginate (SA) film was engineered via drop-casting [173]. Molecular dynamics and DFT simulations revealed KA-ACN/HPMC-SA’s strong binding with trimethylamine (TMA), while UV–vis studies confirmed KA inhibits ACN deprotonation and promotes hydration equilibrium. The optimized 1KA-ACN/HPMC-SA sensor achieved a 15.79 μM LOD for TMA and displayed a maximum ΔE of 31.28 ± 0.76 during salmon spoilage at 4 °C after 10 days [61]. Future development should focus on optimizing material hydrophobicity through additives such as beeswax, extending applicability to other protein-rich foods including meat and dairy products, and integrating smartphone applications for quantitative analysis (Figure 7A). Separately, rare-earth hybrid composites (CD-LEuH@MnO_2_) utilized MnO_2_’s oxidase-like activity to catalyze H_2_O_2_-free TMB oxidation (linear range: 0.5–4.5 μM, LOD: 0.064 μM) [62]. This method offers significant advantages including hydrogen peroxide-free operation through direct TMB oxidation catalyzed by MnO_2_, which simplifies the analytical procedure. It simultaneously achieves high sensitivity, strong interference resistance, and rapid response. However, limitations encompass complex material synthesis involving multi-step hydrothermal/exfoliation/assembly procedures with total duration exceeding 24 h, instrumentation dependency requiring fluorescence/UV spectrophotometers that hinder field deployment, and elevated costs of rare-earth components where expensive europium salts restrict large-scale applications (Figure 7B). The experimental approach for nanoparticle-based colorimetric sensor arrays primarily utilizes the silver mirror reaction. Aldehydes in baijiu reduce Ag^+^ ions adsorbed on nanoparticle surfaces (Au/Ag NPs) to Ag^0^ atoms, which subsequently deposit to form core–shell nanostructures designated as Au@Ag or Ag@Ag. This induces distinct color changes, such as the transition of Au NPs from red to brown. A sensor array was then constructed using four nanoparticle variants: 5 nm and 13 nm Au NPs, alongside 5 nm and 15 nm Ag NPs, to amplify differential responses to various aldehydes. Following this, ΔRGB values extracted before and after reactions generated fingerprint patterns. These patterns enabled classification through pattern recognition techniques including hierarchical cluster analysis and linear discriminant analysis. This method achieves linear formaldehyde detection within 0.05–50,000 μM, with an impressively low limit of detection (LOD) of 0.02 μM. Key advantages include the following: instrument-free operation through visual colorimetric assessment, enhanced by smartphone or RGB analysis; rapid and economical analysis by eliminating pretreatment steps compared to traditional chromatographic/spectroscopic methods; and multi-target capability allowing simultaneous aldehyde discrimination, formaldehyde quantification, and baijiu brand identification within a single array. However, limitations encompass potential nanoparticle destabilization by high ethanol concentrations, subjectivity in visual interpretation under variable lighting, batch-to-batch variations in nanoparticle synthesis affecting reproducibility, and exclusive reliance on aldehydes while neglecting critical flavor components such as acids and esters. Future refinements should focus on enhancing interference resistance and implementing standardized lighting or smartphone-assisted quantification protocols [63] (Figure 7C). This novel colorimetric sensor, comprising bimetallic Ag_2_CrO_4_ nanoparticles (NPs), enables rapid, highly sensitive, and selective detection of the organophosphorus pesticide dimethoate (DMT) in vegetables. The catalytic mechanism operates through dual enzymatic pathways: (1) oxidase-like activity, generating superoxide radicals (·O_2_^−^) and singlet oxygen (^1^O_2_) from dissolved oxygen to oxidize TMB; and (2) laccase-like activity: catalyzing 2,4-DP to form semiquinone radicals that subsequently condense with 4-AAP. The method achieves impressive LOD at 8.7 μg/L via the oxidase pathway and 10.9 μg/L through the laccase pathway. Key advantages include dual-signal channel verification—where oxidase-mediated blue intensification cross-validates laccase-triggered red attenuation to significantly enhance accuracy—and naked-eye discernibility of color changes at DMT concentrations exceeding 40 μg/L without requiring sophisticated instrumentation. However, limitations encompass environmental susceptibility where high salinity, extreme temperatures, or pH variations may impair catalytic activity, alongside constraints in real-sample adaptability. Future development should focus on implementing protective coatings, optimizing nanostructural design, and streamlining sample pretreatment protocols [64,174] (Figure 7D).

### 4.3. Fundamental Recognition Principles

The fundamental recognition principles in colorimetric sensing rely on target–probe interactions triggering macroscopic color changes through four synergistic mechanisms [175]: chemical binding achieves molecular locking via coordination—exemplified by Fe^2+^-phenanthroline complexation forming orange-red complexes at 0.1 μM LOD [176]—covalent bonding through maleimide-thiol addition, and redox reactions like Fe_3_O_4_ nanozyme-catalyzed TMB oxidation to blue products; physical effects amplify signals through localized surface plasmon resonance shifts such as AuNP aggregation causing red-to-purple transitions for 0.1 ng/mL toxin detection [177], surface energy transfer in quantum dot-graphene systems, and π-π stacking enabling pyrene excimer-based 0.02 ppb PAH sensing [178]. Biomolecular recognition enhances selectivity through four primary pathways: enzyme-substrate cascades—specifically glucose oxidase-HRP-TMB systems [179]—antibody-antigen binding that detects E. coli O157 at 10^3^ CFU/mL, aptamer conformational switching, and molecularly imprinted cavities achieving over 95% tetracycline recovery. Stimuli-responsive recognition enables environmental adaptation through pH-sensitive anthocyanins exhibiting reversible red-blue transitions for freshness indication, thermo-sensitive hydrogels like PNIPAM-modulated Au nanorods achieving 80 nm localized surface plasmon resonance shifts, and gas-responsive porphyrin membranes undergoing amine-triggered green-to-red spectral transitions [180,181]. These principles overcome sensitivity boundaries via nanozyme cascade amplification providing 50-fold signal enhancement; CRISPR-colorimetry utilizing Cas12a cleavage-induced AuNP aggregation for single-virus detection [182,183]; and ratiometric outputs maintaining dual-probe absorbance ratio errors below 5%—collectively achieving detection limits spanning pM-level noble metal systems to CRISPR-enabled single-molecule resolution. When integrated with smartphone RGB analysis for on-site pesticide detection at 8.7 μg/L [184] and microfluidic array multiplexing, they establish a molecular interaction-optical conversion-intelligent analysis paradigm applicable to food safety monitoring (0.01 ppm organophosphorus), environmental Hg^2+^ tracking (0.5 nM), and medical diagnostics. Field-deployable advantages including under five-minute response times and visual readout capabilities constitute core competitiveness [185,186,187].

## 5. Electrochemical Sensing

### 5.1. Signal Output Behavior

Electrochemical sensing employs six core signal output modalities: Amperometric output quantifies targets via redox current changes under constant potential—exemplified by glucose oxidase generating current on Pt microelectrodes achieving 0.1 μM detection limits [188,189] and Pt@Fe_3_O_4_ nanozymes amplifying H_2_O_2_ reduction signals 50-fold for 0.01 ppm pesticide detection [190]. Potentiometric output relies on logarithmic interfacial potential responses including ion-selective electrodes with Nernstian 59 mV/decade slopes for K^+^ monitoring and FET charge modulation enabling 0.01-unit pH resolution [191,192]. Impedimetric output enables label-free real-time monitoring through charge transfer resistance changes, demonstrated by 200 Ω ΔRct increases during E. coli adhesion at 10^2^ CFU/mL and nano-antibody complexes achieving 0.1 pg/mL aflatoxin sensitivity [193,194]. Conductometric/capacitive output leverages solution conductivity shifts for bacterial proliferation monitoring at 10^3^ CFU/mL or electric double-layer capacitance variations enabling femtomolar protein detection. Photoelectrochemical output utilizes photoexcited semiconductor amplification—illustrated by CdS/TiO_2_ heterojunctions boosting photocurrent 20-fold for 0.3 pM microcystin detection [195,196] with upconversion material-enhanced tissue penetration. Field-effect transistor output modulates channel conductivity through aptamer-functionalized graphene FET Dirac point shifts detecting 1 nM ATP [197] or Hg^2+^ adsorption n-doping in MoS_2_ FET at 0.1 nM detection limits. These modalities advance through multichannel arrays enabling 16-electrode synchronization with machine learning classification exceeding 95% accuracy for heavy metals, wearable flexible devices exemplified by screen-printed electrodes monitoring sweat lactate within 30 s [198], and self-powered systems utilizing microbial fuel cells converting biochemical oxygen demand to current—collectively pushing detection limits from picomolar to zeptomole levels (10^−21^ mol) in food safety monitoring detecting nitrite at 0.1 μM, environmental Hg^2+^ tracking at 0.01 ppb, and medical neural signal acquisition. Future innovations focus on CRISPR-electrochemistry with Cas12a cleavage-triggered electron transfer for single-virus detection and biodegradable in vivo devices employing Zn electrodes enabling 72-h antibiotic metabolism monitoring.

### 5.2. Electrochemical Sensing Leverages Diverse Nanomaterials

**Figure 8:**
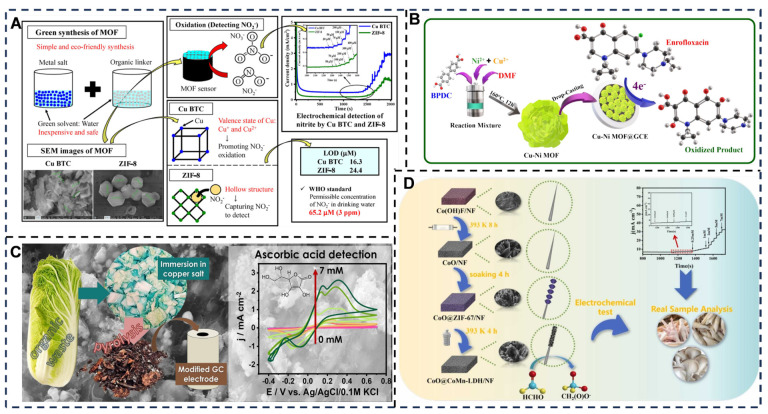
(**A**) Electrochemical sensing of nitrite by Cu and Zn based metal–organic frameworks—a green synthesis approach. Adapted with permission from Ref. [65]. Copyright 2025 Elsevier. (**B**) Illustration of the synthesis of Cu-Ni-MOF and their attachment on GC electrode for the sensing of EF. Adapted with permission from Ref. [66]. Copyright 2025 Elsevier. (**C**) Transforming organic waste: Cabbage-derived carbon containing copper for electrochemical sensing of ascorbic acid. Adapted with permission from Ref. [67]. Copyright 2025 Elsevier. (**D**) A novel self-supporting coral-like CoO@CoMn-LDH nanoarray electrocatalyst derived from ZIF with hierarchical structure exhibits excellent sensing performance for formaldehyde in food samples. Adapted with permission from Ref. [68]. Copyright 2025 Elsevier.

In the field of electrochemical sensing regarding MOFs, copper-based (Cu) and zinc-based (Zn) MOFs were utilized for the electrochemical detection of nitrite ions [199,200]. The synthesized Cu BTC (BTC: 1,3,5-benzenetricarboxylic acid) and ZIF-8 (zeolitic imidazolate framework-8) were characterized via XRD, SEM, Fourier transform infrared spectroscopy (FT-IR), and X-ray photoelectron spectroscopy (XPS), revealing that Cu BTC exhibited a thick rod-like morphology with aggregation while ZIF-8 displayed a non-aggregated rhombic dodecahedron structure [201,202,203]. Cyclic voltammetry (CV) and chronoamperometry achieved LOD of 16.39 µM and 24.48 µM for nitrite ions, values significantly below the WHO standard of 65.2 µM, with successful validation in tap water samples [204,205]. Concurrently, a Cu-Ni bimetallic MOF (Cu-Ni-MOF)-based platform enabled real-time enrofloxacin (EF) detection; this antimicrobial agent, used for livestock/poultry infections, poses health risks [65] (Figure 8A). The sensor, fabricated by drop-casting Cu-Ni-MOF onto GCE, demonstrated 1.7-fold higher EF oxidation current and enhanced catalytic activity versus parent materials, achieving a linear range of 200 nM–2 mM, LOD of 15.17 nM, and sensitivity of 638.71 µA·mM^−1^·cm^−2^ [206]. Its electrocatalytic synergy arose from Cu^2+^-pyridone/carboxylate interactions and Ni^2+^-facilitated electron transfer [207], validated in milk/egg/water samples via LC-MS, while also exhibiting photocatalytic EF degradation under visible light [66,208] (Figure 8B). Regarding noble-metal elements, carbon sensors derived from Cu(NO_3_)_2_-soaked cabbage midribs via pyrolysis showed optimal performance: ascorbic acid sensitivity of 3.91 μA·μM^−1^·cm^2^, linearity (0.2 μM–7 mM), LOD (0.05 μM), high selectivity in interferant-rich environments, and efficacy in orange juice analysis, confirming upcycled cabbage waste as a low-cost, eco-friendly precursor with dual environmental/biomedical value [67] (Figure 8C). A hierarchically structured, self-supporting coral-like CoO@CoMn-LDH nanoarray was synthesized in situ on nickel foam (NF) via a two-step templating strategy using Co(OH)F nanorods and ZIF-67 precursors. Integration with conductive NF mitigated traditional LDH limitations (low conductivity/agglomeration), significantly boosting specific surface area, active-site density, electron transport, and mass transfer. As a formaldehyde (FA) sensor, it achieved a wide linear range (0.035–7 mM), low LOD (7.8 × 10^−3^ mM, S/N = 3), and precise FA quantification in food samples [68,209,210] (Figure 8D).

**Figure 9:**
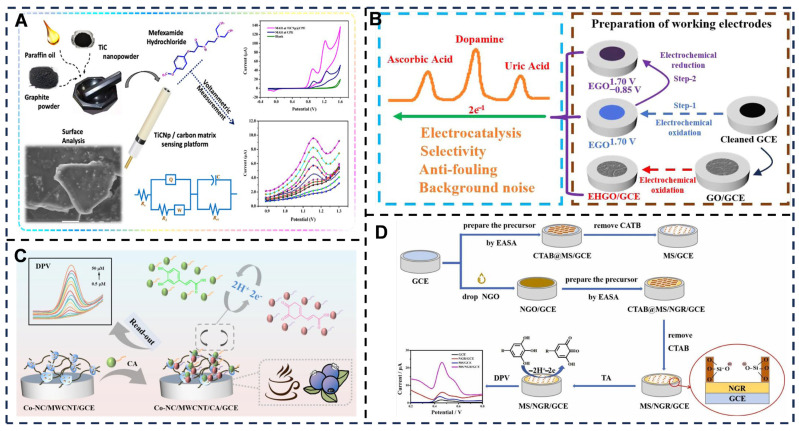
(**A**) Ultra-sensitive platform for the detection of a psychostimulant drug using a nanostructured titanium carbide/carbon matrix: insights into electrochemical sensing mechanism. Adapted with permission from Ref. [69]. Copyright 2025 Elsevier. (**B**) In situ electrochemical fabrication of holey graphene oxide and oxo-functionalized graphene for electrochemical sensing. Adapted with permission from Ref. [212]. Copyright 2025 Elsevier. (**C**) Fabrication of cobalt single-atom catalyst supported on multiwalled carbon nanotubes for enhanced electrochemical sensing of caffeic acid in food samples. Adapted with permission from Ref. [70]. Copyright 2025 Elsevier. (**D**) Schematic illustration of the preparation of MS/NGR/GCE and sensing of TA. Adapted with permission from Ref. [71]. Copyright 2025 Elsevier.

For carbon-based nanomaterials, a nanostructured TiC/carbon matrix sensor detected psychostimulant mefexamide hydrochloride (MAH), exhibiting selective pH 7.0 phosphate buffer response, enhanced electrocatalysis versus bare electrodes, diffusion-controlled redox peaks (two pairs), and SWV-validated linearity (8.0–200.0 μM) with LOD/LOQ of 3.6 × 10^−7^ M/11.9 × 10^−7^ M. Interference studies confirmed simplicity/sensitivity/selectivity for MAH detection in biofluids/water [69,211] (Figure 9A). Li et al. [212] electrochemically generated holey graphene oxide and oxo-functionalized graphene in green mild solution, enabling simultaneous DA/AA/UA detection with optimized electroactivity, antifouling, selectivity, and noise reduction; interfacial oxo-groups/defects critically governed performance, revealing a sensitivity-versus-antifouling/selectivity trade-off (Figure 9B). In composite nanomaterials, Yang et al. [70] developed a Co-NC/MWCNT platform (Co single atoms on N-doped carbon + MWCNTs) for caffeic acid (CA) detection: MWCNTs accelerated electron transfer while Co atoms served as catalytic sites, with electrostatic synergy enhancing redox kinetics [213] (Figure 9C). The sensor achieved 0.162 μM LOD (0.5–50 μM linearity), excellent spike recoveries, anti-interference capability, and accuracy matching standard methods in blueberry/coffee analyses, alongside 86.4–101.0% recoveries and 0.06–3.50% RSD. Wang et al. [71] fabricated MS/NGR/GCE via in situ electrochemistry; its large surface area, high loading capacity, and superior electron transfer enabled dual linear tannin detection ranges (0.10–5.0 μmol/L and 5.0–80 μmol/L) with 0.050 μmol/L LOD (S/N = 3), significantly outperforming single-component modified electrodes (Figure 9D).

### 5.3. Fundamental Recognition Principles

The fundamental recognition principles in electrochemical sensing encompass five core mechanisms [214]: Biomolecular recognition leverages specific binding of enzymes, antibodies, or aptamers for selective detection—exemplified by glucose oxidase catalysis [215]—despite biomolecular stability constraints. Host–guest chemical recognition employs artificial receptors like molecularly imprinted polymers and cyclodextrins achieving stable detection through spatial matching including metal ion chelation [216]. Catalytic recognition utilizes electrocatalytic or enzyme-mimetic capabilities of noble metal nanoparticles and MOFs, detecting targets via reduced overpotential [217] and amplified current as in H_2_O_2_ detection [218]. Interfacial affinity recognition exploits physicochemical adsorption forces such as electrostatic/hydrophobic interactions and π-π stacking [219,220] on functionalized COOH/NH_2_-surfaces [221] for selective enrichment demonstrated by dopamine adsorption on carbon nanotubes [222,223]. M ass-transport-controlled recognition employs selective barriers including Nafion membranes and MOF pores [224,225] regulating mass transfer via diffusion kinetics i_lim_ = nFADC/δ, enabling anti-interference detection through molecular sieving as with ZIF-8 filtering H_2_O_2_ [226]. Modern sensors integrate these principles [227], such as aptamer-functionalized MOFs combining biorecognition with catalytic amplification [228,229,230], to overcome individual limitations and enhance sensitivity, selectivity, and stability.

## 6. Pretreatment and Analytical Procedures Prior to Sample Detection Across Four Sensing Modalities

In the aforementioned study utilizing fluorescence probes for VB_2_ detection: for orange juice beverage pretreatment, samples underwent centrifugation at 8000 rpm for 10 min to remove suspended particles, followed by filtration through a 0.22 μm membrane to eliminate residual impurities [231]. The filtrate was subsequently diluted with 10 mM PBS buffer adjusted to pH 5.0 to achieve appropriate analyte concentrations. Milk samples were processed through dilution and protein/fat precipitation. This precipitation involved sequential addition of 3 mL ethanol to disrupt fat structures, 3 mL of 1% potassium oxalate solution to precipitate calcium ions, and 1 mL of 20% zinc acetate solution to precipitate proteins [232]. The treated milk was then filtered and diluted. To enhance detection sensitivity in juice analysis, solid-phase extraction using C18 columns or vacuum freeze concentration may be implemented [45]. For milk processing, ultrafiltration centrifugation with a 3 kDa molecular weight cutoff could replace chemical precipitation to minimize riboflavin loss. In the literature concerning fluorescent nanosensor detection of multiple tetracycline antibiotics, food matrices undergo a four-stage pretreatment protocol: homogenization of 0.1 g pulverized samples from fish or pork or 10 mL milk with 5 mL aqueous solution containing 50 μL trichloroacetic acid; centrifugal separation at 10,000 rpm for 10 min after 5-min homogenization, followed by supernatant collection; pH adjustment of the supernatant to 7.0 using 1 M sodium hydroxide; and membrane filtration through 0.22 μm filters with subsequent dilution of the filtrate for analysis [233]. Trichloroacetic acid functions as a protein precipitant to mitigate matrix interference. This sample preparation methodology could be enhanced through three strategic modifications: substituting trichloroacetic acid with sulfosalicylic acid due to its milder acidity that reduces tetracycline degradation; implementing MOF-functionalized solid-phase extraction cartridges to leverage selective adsorption of tetracyclines via specific β-diketone structural binding while eliminating lipid and pigment interference; and simplifying pH adjustment by employing buffered precipitating agents including phosphate-buffered trichloroacetic acid solutions pre-adjusted to pH 7.0 [49].

In Raman sensing detection of thiram, food matrices enable direct analysis without any pretreatment. For Raman sensing detection of antibiotic residues in eggs, egg white samples undergo a sequential pretreatment protocol comprising three critical steps: precipitation and impurity removal through mixing with an acetonitrile-water mixture in a 2:1 volume ratio followed by ultrasonication and centrifugation, with subsequent concentration of the supernatant to solid residues at 60 °C [53]; ethanol extraction via dispersion of solid residues in anhydrous ethanol under ultrasonication and centrifugal collection of the supernatant; and final filtration purification [233]. To enhance analytical efficiency, this methodology could be strategically refined through streamlined impurity removal via the QuEChERS method employing single-step acetonitrile extraction combined with PSA and C18 sorbent purification; miniaturized pretreatment using microextraction techniques such as SPME or magnetic solid-phase extraction to reduce solvent consumption; and incorporation of enzymatic digestion to facilitate protein decomposition [152].

For colorimetric sensing detection of pesticide residues in food samples, the analytical protocol involves repeated washing with sodium acetate buffer adjusted to pH 4.0 and HEPES buffer at pH 8.0, followed by direct detection post-filtration without requiring concentration [234]. Baijiu matrices enable immediate analysis without pretreatment. In salmon matrix detection, sample processing entails portioning through cutting, co-sealing with sensors, and refrigerated monitoring at 4 °C [58]. For electrochemical detection of caffeic acid in blueberry matrices, samples undergo sequential pretreatment beginning with ethanol addition followed by ultrasonication. The resultant mixture is then subjected to centrifugal concentration for two hours. The supernatant subsequently undergoes filtration to obtain the test solution. This methodology could be enhanced by integrating solid-phase extraction techniques, specifically utilizing C18 or hydrophilic adsorbent SPE cartridges to concentrate caffeic acid [70]. Such modification would improve analyte recovery while removing interferents including pigments and sugars, thereby enhancing interference resistance (Table 2).

## 7. Biosensing Application

Detection of chemical hazards employs a complementary network of fluorescent [233], colorimetric [235], Raman [236], and electrochemical [237] sensing for food safety monitoring [238]. Fluorescent sensing dominates ultra-trace biological toxin detection with zeptomolar sensitivity [239], exemplified by aptamer-quantum dot probes achieving 0.003 ng/mL detection limits for aflatoxin B1 [240,241], while enabling dynamic heavy metal tracking such as Hg^2+^ via gold nanocluster quenching [242]. Colorimetric sensing supports on-site preliminary screening through visual readability and ultra-low cost—under $0.1 per test strip [234]—demonstrated by acetylcholinesterase-TMB chromogenic systems for organophosphorus pesticides and Sudan I-induced gold nanoparticle aggregation achieving 10 ppb detection limits [243,244,245]. Raman sensing confirms pesticide residues via molecular fingerprints including chlorpyrifos’ characteristic 657 cm^−1^ peak with SERS enhancement using gold nanostar dimers [246], and identifies recycled cooking oil adulteration through abnormal cholesterol peaks at 1440 cm^−1^ [247,248,249]. Electrochemical sensing delivers precise quantification, detecting enrofloxacin at 15.17 nM limits using Cu-Ni-MOF electrodes and nitrites via nanozyme-catalyzed responses within 3 s, serving as the core method for additive and heavy metal analysis [250,251].

Detection of biological hazards employs a multilayered defense system for foodborne pathogens using four complementary technologies [252]. Raman sensing achieves single-cell detection of *E. coli* O157:H7 at 1 CFU/mL limits using immunomagnetic SERS tags [253], while simultaneously identifying parasite eggs through molecular fingerprints such as *Trichinella* larval cyst wall features [254]. Colorimetric sensing enables rapid preliminary screening of *Salmonella* visible to naked eye at 10^4^ CFU/mL via nanozyme-catalyzed chromogenic reactions—specifically Fe_3_O_4_ nanoparticle-H_2_O_2_-TMB systems [255,256]. Electrochemical sensing coupled with CRISPR-Cas12a achieves single-copy norovirus identification, while fluorescent sensing utilizes quantum dot-labeled antibodies for high-contrast imaging of *Clonorchis* eggs [257,258]. For real-time microbial monitoring, flexible electrochemical electrodes track live microbe electrogenic signals to warn of food spoilage risks.

Control of physical and emerging hazards leverages multi-sensing technologies to address complex challenges like food adulteration and packaging migrants. Raman sensing locates DEHP plasticizer migration hotspots in packaging through 3D imaging at 1 μm resolution and identifies recycled cooking oil adulteration via cholesterol fingerprint peaks at 1440 cm^−1^ [259,260]. Fluorescent sensing employs quantum dot-encoded multiplex detection for honey-adulterating sugars, while colorimetric sensing utilizes pH-responsive hydrogel patches to visually indicate meat spoilage through volatile amine response within 5 min [261,262,263]. For emerging nanopollutants such as TiO_2_ food additives [264], Raman-SERS integration analyzes particle surface chemistry, and electrochemical sensing monitors Ag^+^ dissolution from nanoparticles via impedance changes [265]. Smart freshness monitoring incorporates wearable dual-mode patches integrating colorimetric hydrogels with electrochemical sensors for full-cycle quality tracking of chilled foods [266].

Technological synergy and intelligent advancement enable deep collaboration through a screening-quantification-confirmation-verification chain: Colorimetric test strips preliminarily screen pesticide-positive samples, followed by SERS-Raman validation of aflatoxin with false positive rates below 0.1% [267]; electrochemical sensors rapidly quantify antibiotic residues while fluorescent aptamer probes conduct trace verification achieving 98–102% recovery rates [268]. Intelligent integration includes microfluidic chips combining colorimetric readouts, screen-printed electrodes, and SERS zones enabling 5 samples/minute throughput [269], alongside AI-driven arrays classifying pathogens exceeding 99% accuracy via machine learning of Raman/fluorescent signatures [270]. Future advancements will employ noble metal-perovskite hybrids—exemplified by Au/CsPbBr_3_ enabling 10^3^-fold plasmon-exciton enhanced SERS—and DNA hydrogel-controlled probes for in vivo toxin monitoring, establishing an end-to-end ecosystem from electrochemical heavy metal monitoring in farmland soils to wearable freshness patches on dining tables [271,272,273].

## 8. Summary and Outlook

Future advancements in food safety detection will undergo comprehensive innovation and systemic transformation, with multimodal data fusion and adaptive AI systems serving as core drivers. For instance, integrating multidimensional information—including hyperspectral imaging for capturing surface coloration and internal defects, near-infrared spectroscopy for sugar distribution analysis, and electronic noses for volatile maturity markers—through advanced algorithms such as Transformer architectures or graph neural networks enables robust assessment models. These effectively overcome matrix complexity and environmental variability, precisely evaluating perishable agricultural products like mangoes for ripeness and internal quality. Coupled with dynamic learning algorithms that adapt to geographical and seasonal heterogeneity, this approach significantly enhances detection accuracy and timeliness. Synergistic innovations in emerging AI frameworks and high-performance nanomaterials will invigorate detection technologies. Graph convolutional networks process unstructured data by converting disease spots or cracks on apple surfaces into knowledge graphs, enabling precise pathogen differentiation between conditions like black spot and anthracnose. Autoencoders integrated with convolutional neural networks efficiently extract spectral features, amplifying adulterant signals during olive oil authenticity verification against adulterants such as low-grade oils. Material innovations include MOF-quantum dot composites that enhance fluorescence sensitivity for trace aflatoxin B1 detection in peanuts, and Fe_3_O_4_ nanozymes mimicking peroxidase activity to replace natural enzymes—improving stability and efficiency in milk antibiotic residue assays. Surface-modified nitrocellulose membranes or ZIF-8-functionalized banana paper further enable cost-effective, high-performance freshness test strips for meat. Multimodal signal outputs with cross-validation mechanisms substantially reduce false positives, exemplified by simultaneous colorimetric changes and fluorescence enhancement to confirm melamine in milk [274,275].

Future research must prioritize developing intelligent, integrated next-generation monitoring systems by focusing on four critical frontiers: high-throughput multiplex detection through microfluidic platforms enabling automated parallel screening of pathogens or pesticide residues in single samples; on-site analysis via seamless sensor-smart device integration using smartphone-based test strip quantification or wearable monitors for real-time freshness tracking in cold-chain foods; recognition element innovation employing engineered aptamers and CRISPR-Cas systems to achieve specific mycotoxin capture in complex juices and authenticate meat speciation; and sustainable advanced materials development including DNA hydrogels for lead detection in water alongside noble metal-perovskite hybrids that enhance electrochemical pesticide sensors. Critically, AI-powered data analytics will decode multisensory signals while identifying multidimensional risk patterns, enabling predictive management. The ultimate goal is to overcome core challenges—particularly matrix compatibility barriers and real-time hazard recognition—establishing end-to-end intelligent detection ecosystems spanning “from agricultural soil to consumer tables.” This includes electrochemical sensors for in situ cadmium monitoring and intelligent packaging indicating real-time freshness of fresh-cut produce, thereby delivering unprecedented global food safety assurance [276,277,278,279].

## Figures and Tables

**Table 1:** Summary of LODs in nanostructure-engineered biosensing for food safety.

Sensing Type	Target Analyte	Signal Mechanism	Nanomaterial	LOD	SERS
Fluorescence Sensing					
	*E. coli*	On-off-on fluorescence	Au NCs	89 CFU/mL	[44]
	VB_2_	Ratiometric fluorescence	SiNPs	135 nM	[45]
	S^2−^	Fluorescence quenching	F-SiNPs	0.1 μM	[46]
	UO_2_^2+^	DNAzyme/GO nanosystem	UCNPs	25 pM	[47]
	TCs	Ratiometric fluorescence	HC@Eu-MOF	4.8 nM (OTC); 16.5–56.4 nM (other TCs)	[48]
	Foodborne pathogens	Turn-on fluorescence	Fe-MOF	0.464 log CFU/mL (*S. aureus*); 0.584 log CFU/mL (*E. coli*)	[49]
Raman Sensing					
	Methylene blue, thiabendazole	SERS intensity	Au nanostar monolayer	4.2 × 10^−12^ M	[50]
	Thiram	SERS intensity	Au@Ag core–shell nanoflowers	7.09 × 10^−8^ mol/L	[51]
	ciprofloxacin	Dual-resonance SERS	V_5_S_4_ nanopompons	10^−7^ M	[52]
	tetracycline	Dual-resonance SERS	V_5_S_4_ nanopompons	10^−8^ M	[52]
	chloromycin	Dual-resonance SERS	V_5_S_4_ nanopompons	10^−7^ M	[52]
	Antibiotic residues	SERS intensity	TiO_2_/ZnO heterostructure	3.94 × 10^−8^ M	[53]
	PATP (model molecule)	SERS intensity	AgNWs@MOF	10^−13^ mol/L (liquid); 10^−7^ mol/L (gas)	[54]
	Wheat gluten	SERS intensity	AgNPs/MOF substrate	1.16 × 10^−16^ mol/L	[55]
	Carbendazim (pesticide)	SERS intensity	Ag/CuO nanocomposite	8.85 × 10^−11^ M	[56]
Colorimetric Sensing					
	Ascorbic acid	Oxidase-like activity	Fe_3_O_4_@SiO_2_@NiCo_2_S_4_	0.36 μM	[57]
	Sarcosine	Oxidase-like activity	Fe_3_O_4_@SiO_2_@NiCo_2_S_4_	0.42 μM	[57]
	Lactose	Peroxidase-like activity	Cu/N-doped carbon nanozyme	0.03 mM	[58]
	β-Galactosidase	Peroxidase-like activity	Cu/NC NS	0.01 U/mL	[58]
	ferulic acid	Peroxidase-like activity	Ta-MOF	0.19 μM	[59]
	tannic acid	Peroxidase-like activity	Ta-MOF	0.06 μM	[59]
	chlorogenic acid	Peroxidase-like activity	Ta-MOF	0.11 μM	[59]
	Nandrolone	Aptamer binding	Zn-Fe MOF	0.85 nM	[60]
	Trimethylamine	Pigment binding	KA-ACN/HPMC-SA film	15.79 μM	[61]
	Ascorbic acid	Oxidase-like activity	CD-LEuH@MnO_2_	0.064 μM	[62]
	Formaldehyde	Nanoparticle color shift	Au/Ag NPs	0.02 μM	[63]
	Dimethoate (pesticide)	Dual-enzyme mimic	Ag_2_CrO_4_ NPs	8.7 μg/L (oxidase); 10.9 μg/L (laccase)	[64]
Electrochemical Sensing					
	Nitrite	Electrocatalysis	Cu BTC MOF; ZIF-8 MOF	16.39 μM; 24.48 μM	[65]
	Enrofloxacin	Oxidation current	Cu-Ni-MOF	15.17 nM	[66]
	Ascorbic acid	Electrocatalysis	Cabbage-derived carbon/Cu	0.05 μM	[67]
	Formaldehyde	Electrocatalysis	CoO@CoMn-LDH/NF	7.8 × 10^−3^ mM	[68]
	Mefexamide hydrochloride	Redox peaks	TiC/carbon matrix	3.6 × 10^−7^ M	[69]
	Caffeic acid	Redox catalysis	Co-NC/MWCNT	0.162 μM	[70]
	Caffeic acid	Electrocatalysis	MS/NGR	0.05 μmol/L	[71]

**Table 2:** Detection methods and corresponding target substances/pollutants in food safety biosensing.

Detection Method	Target Substance/Pollutant	Example Application	Key Nanomaterial/Technology	Food Matrix	SERS
Fluorescence Sensing					
	Heavy metals (e.g., Hg^2+^)	Real-time monitoring in water and agricultural products	Gold nanoclusters	Water, fish, grains	[44]
	Biological toxins (e.g., aflatoxin B1)	Mycotoxin screening in nuts and cereals	Quantum dot-labeled antibodies	Peanuts, maize	[46,105]
	Antibiotics (e.g., tetracycline)	Veterinary drug residue detection in animal-derived foods	HC@Eu-MOF nanosensor	Milk, meat	[48]
	Pathogens (e.g., *E. coli*, *S. aureus*)	Rapid bacterial detection in dairy and beverages	Fe-MOF	Milk, juice	[49]
	Vitamins (e.g., riboflavin, VB_2_)	Nutrient quantification in fortified foods	Silicon nanoparticles	Juices, dairy products	[45]
	Uranyl ions (UO_2_^2+^)	Contaminant tracking in water sources	Upconversion nanoparticles with DNAzyme/GO	Drinking water	[47]
Raman Sensing					
	Pesticides (e.g., chlorpyrifos, thiram)	Residue screening in fruits and vegetables	Au nanostar dimers; Au@Ag core–shell nanoflowers	Apples, milk	[50,51]
	Antibiotics (e.g., ciprofloxacin, tetracycline)	Drug residue identification in animal products	V_5_S_4_ nanopompons; TiO_2_/ZnO heterostructure	Eggs, meat	[52,53]
	Allergens (e.g., wheat gluten)	Gluten detection in processed foods	AgNPs/MOF substrate	Soy sauce, vinegar	[55]
	Additives (e.g., Sudan dyes)	Illegal dye identification in spices	Ag/CuO nanocomposite	Tea leaves, chili powder	[56]
	Recycled cooking oil adulterants	Cholesterol-based authentication in oils	Gold nanostar monolayers	Cooking oils	[54]
Colorimetric Sensing					
	Antioxidants (e.g., ascorbic acid)	Quality control in beverages and supplements	Fe_3_O_4_@SiO_2_@NiCo_2_S_4_ nanocomposites	Juices, vitamin tablets	[57]
	Hormones/illegal additives (e.g., nandrolone)	Doping agent detection in sports foods	Zn-Fe MOF aptasensor	Meat products, supplements	[60]
	Pesticides (e.g., dimethoate)	On-site residue testing in vegetables	Ag_2_CrO_4_ nanoparticles	Leafy greens, tomatoes	[64]
	Freshness indicators (e.g., trimethylamine)	Spoilage monitoring in seafood and meat	KA-ACN/HPMC-SA film	Salmon, beef	[61]
	Flavors/aldehydes (e.g., formaldehyde)	Authenticity assessment in alcoholic beverages	Au/Ag nanoparticle array	Baijiu, wines	[63]
Electrochemical Sensing					
	Nitrites	Preservative analysis in processed meats and water	Cu BTC/ZIF-8 MOFs	Tap water, cured meats	[65]
	Antibiotics (e.g., enrofloxacin)	Veterinary drug monitoring in dairy and eggs	Cu-Ni-MOF	Milk, eggs	[66]
	Organic acids (e.g., ascorbic acid)	Nutrient and additive quantification in fruits	Cabbage-derived carbon/Cu	Oranges, berries	[67]
	Aldehydes (e.g., formaldehyde)	Contaminant detection in preserved foods	CoO@CoMn-LDH nanoarray	Fish, processed foods	[68]
	Phenolic compounds (e.g., caffeic acid)	Antioxidant screening in functional foods	Co-NC/MWCNT	Blueberries, coffee	[70,71]

## Data Availability

No new data were created or analyzed in this study. Data sharing is not applicable to this article.
